# The “PPI Hawker”: an innovative method for patient and public involvement (PPI) in health research

**DOI:** 10.1186/s40900-020-00205-6

**Published:** 2020-06-16

**Authors:** L. Luna Puerta, H. E. Smith

**Affiliations:** 1 Family Medicine and Primary Care, Lee Kong Chian School of Medicine, Nanyang Technological University Singapore, Singapore, 308232 Singapore; 2 Division of Public Health and Primary Care, Brighton and Sussex Medical School, Brighton, BN1 9PH UK

**Keywords:** Community-based methods, Collaborative research, Empowerment, Health research, Participatory research, Patient and public involvement and engagement

## Abstract

**Background:**

Patient and Public Involvement (PPI) in health research entails doing research ‘with’ the public. Successful PPI requires a diversity of patients’ perspectives and experiences. In Singapore, including the public’s voice in research is still in its infancy and different ways of involving the public have to be explored.

Our aims were to describe a PPI initiative that enables members of the public to share their ideas and opinions about health research, and to assess the feasibility, accessibility and utility of the initiative.

**Methods:**

Building on the concept of the PPI Café used in the west we designed a “PPI Hawker” for Singapore. Here Hawker Centres rather than cafes are used frequently for eating and socialising, providing a one-stop destination for a wide section of society. The PPI facilitators approached people sitting at tables and joined them to discuss questions of relevance to a local research study. Observations and reflexive field notes were used to evaluate the “PPI Hawker’s” feasibility, acceptability and utility in the Singaporean community.

**Results:**

In three “PPI Hawkers” we approached 96 people and 72 (75%) engaged in discussions about the design of a population-based research study. The majority (75%) of participants willingly discussed all of the questions posed to them by the researchers, indicating the feasibility of PPI. The PPI participants came from the three major ethnic groups in Singapore and appeared to be broad in age, suggesting “PPI Hawkers” are easily accessible. Both participants and researchers recognised the utility of the “PPI Hawker”, reflecting on people’s willingness to talk about the research issues, engaging in informative conversations and posing relevant questions.

**Conclusion:**

The “PPI Hawkers” succeeded in engaging the public in conversations about a local population-based study. The public brought to the researchers’ attention a variety of previously unheard perspectives about the research. Each event fostered connectivity between professionals and the public, generating among researchers a more positive perception of the power of public involvement.

“PPI Hawkers” provide an opportunity for co-informed conduct of research studies with diverse members of the public. They create a focus within a community setting for researchers to engage with the public. The resources needed (costs and preparatory time) are relatively few. Not only do “PPI Hawkers” have potential in Singapore, but also for the rest of Asia.

## Plain English summary

Public involvement in research is defined as research being carried out ‘with’ or ‘by’ members of the public rather than ‘to’, ‘about’ or ‘for’ them. The aim of this paper is to describe and evaluate a new public involvement initiative, the “PPI Hawker”. This event creates a space in an Asian context for the public to comment on health-related research.

“PPI Hawkers” are a modification for Singapore of the PPI Café where the public are invited by “baristas” (researchers, public facilitators and PPI facilitators) to enjoy a drink in exchange for commenting on research issues about which the public’s perceptions and comments were needed. The PPI Cafés ‘guests’, write down their comments and these are collected for later analysis. In Singapore, hawker centres, also known as food courts, are used more than cafés, and are frequented by a wide range of people. In “PPI Hawkers”, a team of facilitators joined people at their table to discuss questions of relevance to a local research study. During the encounters one facilitator introduced and guided the conversation in the appropriate local language while a second made notes about the discussion.

During three “PPI Hawkers”, we engaged 72 members of the public in discussions about a population-based research study. The people sharing their views came from three different ethnic groups and were from a broad range of ages. Both researchers and the public recognised the usefulness of this method to facilitate informative conversations which highlighted perspectives not previously heard by the researchers.

We were able to demonstrate that “PPI Hawkers” are effective in Singapore for involving lay people in discussions about research. This method facilitates co-informed conduct of research studies with diverse members of the public and requires few resources or preparatory time. We think this method of PPI may work well in other Asian countries too.

## Background

Patient and public involvement (PPI) in research is defined as research that is carried out ‘with’ or ‘by’ members of the public rather than ‘to’, ‘about’ or ‘for’ them. Although PPI is a common component of health research in Western countries, such as the UK, Australia and Canada [1–5], PPI is still in an early stage of development in Asian countries, including Singapore [6]. It is recognised that PPI enhances the quality and relevance of the research, and its impacts are greater when implemented early in the research cycle [7–9]. If Singapore wishes to remain at the forefront of Asian health research, public involvement in health research must be cultivated, with public perspectives being heard and their concerns addressed.

Often when PPI is in its infancy, a few selected public collaborators are invited by the researchers to engage in a two-way conversation [10]. These same individuals may contribute repeatedly during the research process and also return for future studies led by the same team. Whilst this model of involvement is strong on continuity, it generates concerns about tokenism. There is a risk that the public contributor remains as a “thinker at the edges” [11, 12]. Other PPI initiatives strive for greater diversity in involvement, whilst some focus on greater levels of public impact [13, 14]. Public involvement is a complex and diverse endeavour; each PPI approach has its merits and their appropriateness varies according to the characteristics of the study (for example, research question, study design, resources available and skills of lay-researchers [15–18].

Recent PPI developments have focussed on ways to include disadvantaged communities in these “privileged” spaces, recognising it is often vulnerable people who have the greatest health needs [10, 19, 20]. PPI Cafés are an example of such developments, whereby the characteristics of a “science café” and a PPI workshop were blended. A “science café” is where scientists and science professionals are invited to speak in a casual non-academic setting where everyone can learn and discuss the scientific issues of the day in science and technology, generally for the price of a cup of coffee or a glass of wine [21]. In contrast, PPI workshops a formal, organised training sessions for researchers wanting to learn how to involve lay people in their research activities and for members of the public wishing to familiarise themselves with research and PPI [22]. The PPI Cafés borrow from the science café the casual setting and conversational style and from the PPI workshops learning objective to create an opportunity for the public to have learn about PPI as they contribute to the development and refinement of a real-life research project.

The first PPI Café was piloted at the 2018 Imperial Festival [22], a science festival for the public organised by researchers at Imperial College London. The public were invited to join the “baristas” (researchers, public facilitators and PPI facilitators) for a drink in a space customised to look like a café [22]. Tables were dedicated to different research studies, and at each table a facilitator introduced the research study before the researcher asked about their ‘guests’ perceptions and comments on specific issues. Guests were encouraged to write down their comments and leave them in a container in the centre of each table.

**Fig. 1:**
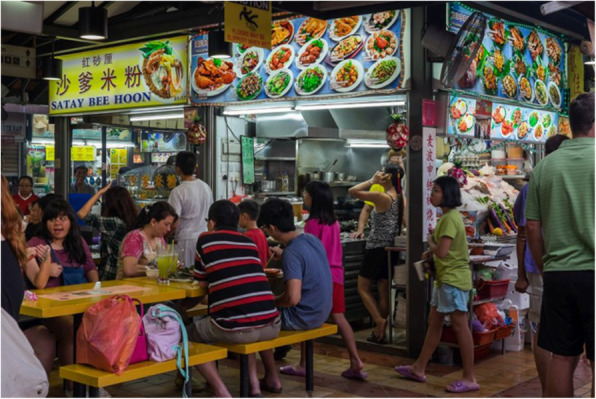
Photograph of Serangoon Gardens Market & Food Centre. Photo Credit: Cynthia Chew [23]

In Singapore cafés are not as common as in Western countries, but hawker centres, also known as food courts, (shown in Fig. 1) are used frequently for eating and socialising. In hawker centres, seating facilities are shared and a complete meal can be enjoyed at one table with each course taken from different vendors. This differs from the Western context, where only food and drink purchased from that vendor can be consumed on the premises exclusive to the vendor. Hawker centres are also more accessible, both physically and financially, to a wider sector of society than cafés. Described as “Singapore in a nutshell” [24], they provide a one-stop destination for everyone, offering a wide choice of high-quality food at inexpensive prices (meals are priced at S$3–4 and a local drink costs about S$1.5 [25]). Open from 7 AM to 10 PM and managed by the National Environment Agency, hawker centres are typically found near public housing estates or transport hubs. The 15,000 food stalls [26] serve the majority of the 5.64 M people living across the country [27]. Hawker centres are so integral to Singaporean life that in March 2019, the Singaporean government nominated hawker culture for UNESCO listing of the Intangible Cultural Heritage of Humanity [28].

To date there has been little attention to the inclusion of PPI in research conducted in Asian countries. Currently in Singapore the role of the public is almost exclusively limited to taking part in research. While the benefits of public involvement and co-production are well established in the literature [7, 29–41], methods for an Asian context have yet to be explored. Here we describe the “PPI Hawker”, an adaptation of the PPI Cafés used in Western cultures. The “PPI Hawker” was designed to enable the Singaporean public to share their personal perspectives relevant to a local population-based study (described in Table 1) in an informal environment throughout the country. This article shares our experiences of developing and conducting “PPI Hawkers”.

**Table 1:** Overview of the population-based study

This population-based study, coordinated by Lee Kong Chian School of Medicine in collaboration with the National Healthcare Group (Singapore) and Imperial College London, was established to identify the genetic and environmental factors that underpin the development of chronic conditions, such as obesity, diabetes, and cardiovascular disease, in Singapore. Ultimately, the goal is to use this knowledge to develop novel ways of predicting, preventing, detecting early and treating chronic diseases in Singapore.	
The genetic and medical predispositions differ between population, and to date similar studies have been conducted in the US, UK and Germany. However, 60% of the world’s population do not have European heritage and this biobank will improve the understanding of disease predispositions in South East Asians. The phenotypic, physiological and biological measurements together with long-term follow-up, will enable the study of the complex interrelationships between environmental, lifestyle and genetic factors on disease risk [42–45]. The study aims to recruit 10,000 Singaporeans aged between 30 to 84 from the three main ethnic groups (Chinese, Malay, and Indian) and others. Each participant will have a comprehensive health screening, including health and lifestyle questionnaires, physical measurements, and extensive physiological and imaging data. After the first visit, participants receive a report detailing important abnormal results.	

## Methods: “PPI Hawker” as a PPI method in health research

### Towards an Asian friendly PPI Café

Conceptually, the “PPI Hawker” was designed to be similar to the PPI Café. We envisioned the “PPI Hawker” would reduce the hierarchies between researchers and the public, enabling a two-way conversation, which could facilitate the sharing of knowledge and improved relevance of research studies. Through short and informal conversations, the “PPI Hawker” was created to bring together members of the community (past, current, and future patients and their caregivers) with researchers, reducing the boundaries between non-experts and experts. The design of “PPI Hawkers” would encourage recognition of complementary expertise by positioning lived experiences and personal perspectives at the same level as expert knowledge. Like for the PPI Café, organising the discussion in a setting familiar to the public would facilitate discussion, encouraging ideas to be explored in spontaneous conversation, building trust between lay people and researchers.

### Designing and testing the feasibility of the “PPI Hawker”

To progress the design of the “PPI Hawker”, we formed a development team with a coordinator, two PPI Leads with experience of conducting PPI Cafés in the UK, a researcher from the population-based study (Table 1) and a Singaporean resident. The coordinator was a Spanish doctoral student, exploring the potential for PPI in Singapore (LLP) and the two PPI Leads were from Imperial College London’s Patient Experience Research Centre (PERC), UK with extensive experience of PPI. Other members of the team were an Asian post-doctoral researchers from the study population-based study and a Singaporean resident, an engineer who was interested in PPI.

Adaptation of the PPI Café to the Singapore context required more than just change in location: the multi-ethnic community required us to consider how we would facilitate, and record discussions conducted in several languages. We decided to work organised in such a way as to maximise the local languages available for discussion (Mandarin Chinese, Cantonese, Malay and Tamil). Note taking was always in English as this is the lingua franca*,* that is the language adopted as a common language between speakers whose native languages are different. By documenting in English, the one common language between the speakers of many different languages in Singapore, our documentation was accessible to all facilitators and researchers.

**Fig. 2:**
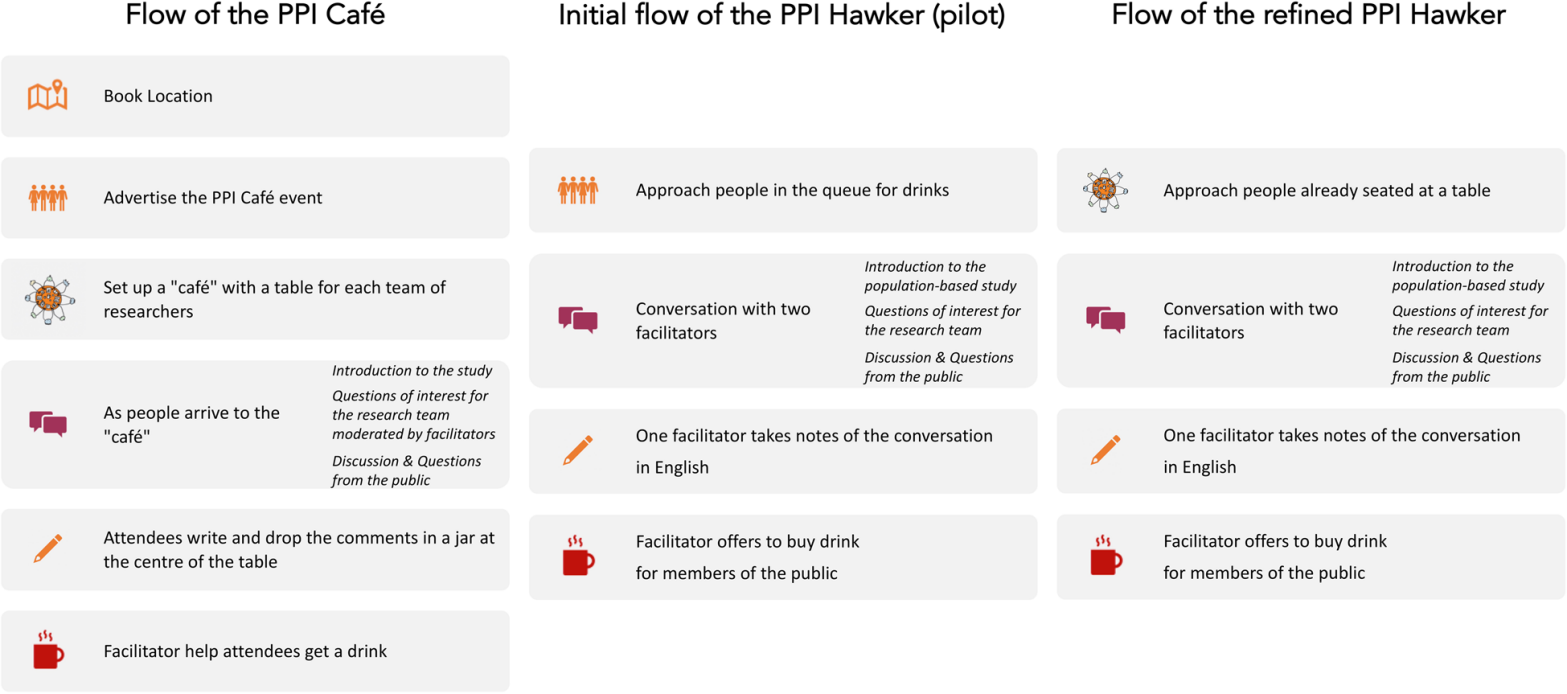
Example of cue card containing a question and prompts for the “PPI Hawkers”

**Fig. 3:**
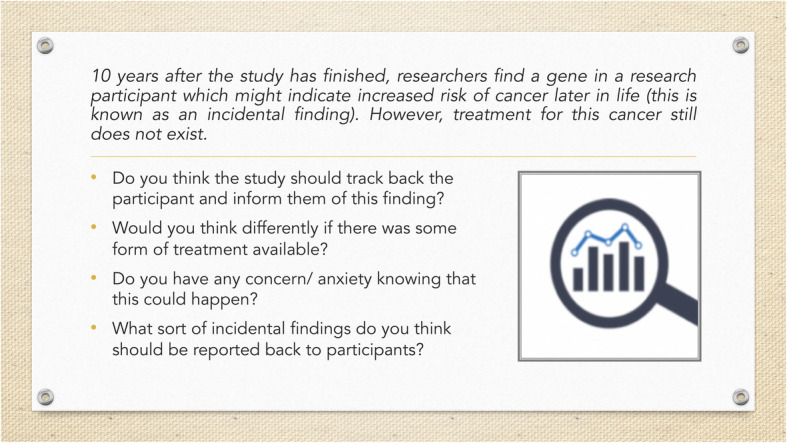
Design of the “PPI Hawker”, building on the PPI Café

In taking the PPI Café (Fig. 2) into the Singaporean context we wished to retain the informal atmosphere but to use the more open, non-institutional and public space of a Hawker Centre. We transferred the idea of using members of the public as facilitators, involving researcher from project, offering participants refreshments (but not making them ourselves), and facilitating in pairs. We dispensed with displaying research materials, but we added the use of cue cards to aid round table discussion (Fig. 3). Participants were also able to refer to a card briefly summarising the study’s aims and methods, to reinforce the initial oral explanation. One facilitator introduced and guided each conversation, using their local language skills whilst the second facilitator documented the discussion.

Having piloted the first “PPI Hawker” as a team of five, we subsequently worked as four (“PPI Hawker” coordinator, researcher from the study, and two public facilitators). The first public facilitator was the same one as in the development team; the second one was an early career scientist who wanted to develop her communication skills with the public and who studied in the same university where LLP is conducting her doctoral studies. None of them had been previously involved in research and they facilitated the “PPI Hawker” on a volunteer non-paid basis. The public facilitators were selected for their language skills (speaking English, and one or more local official languages: Mandarin, Malay and Tamil), their comfort engaging with the public and an interest in research. They were briefed on the research study objectives and the issues the researchers wanted the public to consider. Before going to the Hawker Centres the team used role play to familiarise themselves with the cue cards and how to focus discussion on those topics.

Although LLP coordinated the event, all four facilitators had equal roles and voice during the whole event.

#### A step-by-step guide to the “PPI Hawker”

**Fig. 4:**
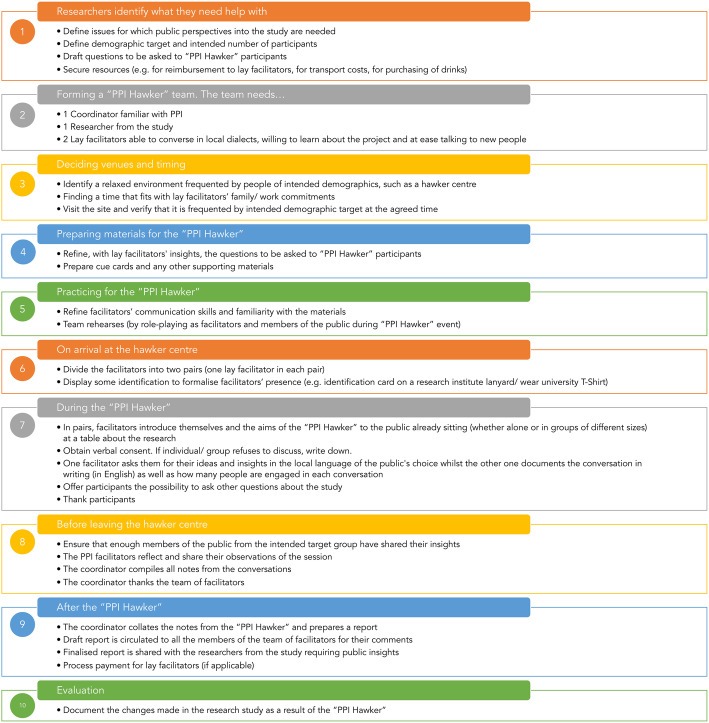
The ten stages in organisation of the “PPI Hawker” (In preparing for this series of 10 stages, attention has been paid to the UK Standards for Public Involvement in Research [46])

The ten stages in organisation of the “PPI Hawker” are summarised in Fig. 4. Such an event starts with a group of researchers willing to seek the lay perspectives in a “PPI Hawker” and to collaborate with a group of facilitators for it. A facilitator familiar with PPI would then work with the study team to come up with topics and questions about which a public perspectives would be valued (Step 1). The facilitation team is then formed, including a representative from the research study and at least two members of the public able to converse in local dialects and comfortable talking to new people (Step 2). Deciding on a suitable venue for the event attention must be paid to the ambience, a relaxed atmosphere in a place frequented by a diverse population and the time envisaged for the “PPI Hawker” (Step 3). As a team the facilitators need to refine the phrasing of the questions (Step 4) and then practice using role play how best to approach people in the hawker (Step 5).

When the “PPI Hawker” event takes place it is advisable for the facilitators to work in pairs (Step 6) to approach people already sitting at a table, either alone or in a group, to introduce them to the study and the aim of the “PPI Hawker”. In our case, the coordinator paired with a lay facilitator, whilst the researcher paired with the second lay facilitator. Once verbal consent is obtained, the discussion of the PPI questions begins with one facilitator managing the discussion and the other documenting the conversation (Step 7). As hawker centres are generally frequented by large crowds, not everyone will be approached, instead the facilitators select based on whether people are already engaged in conversation, appear to be leaving soon or are from the demographic group targeted.

To document the conversations, one of the researchers listed down the main points discussed for each of the questions. After each “PPI Hawker”, the main issues brought up by participants were summarised each facilitator reflected on their interactions with the public, documenting difficulties encountered, for example engaging the public in conversation around a particular aspect of the research (Step 8).

The findings were presented to the research team orally and in a report for them to discuss and agree what adjustments should or could be made to the study (Step 9).

When conducting “PPI Hawkers” the note taking is not routinely verbatim. However in this development and feasibility phase we captured exactly what members of the public offered as feedback on their experience of taking part in the “PPI Hawker”. Facilitators also noted of the number of people sitting at each participating table, together with their observations on demographic characteristics (gender, age and ethnicity) and the attitudes of everyone taking part; this data was used for assessing feasibility but not shared with the population-based study’s researchers.

#### Ethical considerations

In the UK, application for apply for ethical approval to involve the public in the planning or the design stage of research is not necessary [47]. Since Singapore does not yet have guidelines for PPI in research, we followed INVOLVE guidelines from the UK. We did not record any identifying information from “PPI Hawker” participants [48].

#### Criteria for evaluation

Before piloting the “PPI Hawker”, we agreed on the desirable characteristics against which we could evaluate the initiative:

- Feasibility was defined by the capacity to engage members of the public in discussion. Our goals were:
  - ◦ at least 50% people approached agreeing to participate,
  - ◦ to engage a minimum of 20 participants in each session, at least 10 individuals responding to each of the questions posed by the researchers (the questions from each session are listed in Appendices 1 and 2). This criteria was based on sample sizes commonly used in qualitative research [49, 50].
- Acceptability of the “PPI Hawker” was judged by our success engaging with a cross section of the general adult population of Singapore, the target audience for the population based study. We sought representation from diverse ages (20–80) and all major ethnic groups (Chinese, Malay and Indian). To monitor this facilitators made notes about the perceived age of “PPI Hawker” participants and their ethnic group. Acceptability was also assessed by the informal feedback and comments made by participants.
- Utility was the extent to which the public generated ideas were incorporated into the study design by the population-based study’s researchers. Utility was assessed by the researchers’ responses to presentations and written reports about the views elicited by the “PPI Hawkers”.

## Results

### The conduct of three “PPI Hawkers”

#### The “PPI Hawker” pilot

We conducted three “PPI Hawkers”, the first as a pilot to see if we needed to improve on the design. This “PPI Hawker” was held in November 2018, in Serangoon Gardens Market and Food Centre (Fig. 1) and it soon became apparent that inviting participants to join our table as they queued to buy themselves refreshments from the hawker stalls was not going to work (Fig. 2). Everyone approached refused to join our table, but instead invited us to join theirs. After a quick discussion, we decided to approach people already sitting and asking if we could join their table. With this amendment, 28 members of the public participated, but all declined the offer of a free drink.

Our preliminary “PPI Hawker” highlighted the need to be clearer when introducing the concept of PPI and describing the overall aims of the research study to be discussed. The need for this additional information became apparent when some members of the public thought we were trying to recruit them to the study rather than ask for their ideas and opinions. The pilot reinforced the need to work in pairs, with one person documenting the insights shared by members of the public whilst the other facilitated the discussion. To increase the clarity and utility of the documentation we developed a coding system that we cod use in subsequent events.

#### The conduct of two further “PPI Hawkers”

Our first “PPI Hawker” engaged mostly Chinese, so to achieve greater ethnic diversity the two subsequent events were conducted in locations frequented by more Malay and Indian residents (Malay and Indian represent 13 and 9% of Singapore’s population respectively, 74% being Chinese [27]). With our revised method (Fig. 2), we were able to approach a further 49 members of the public of whom we engaged 44 (24 Indian, 13 Malay and 7 Chinese), in short conversations (around 15 min per table) sitting at 33 tables (16 in Admiralty and 17 in Tekka Centre).

### Achieving our goals

The “PPI Hawkers” exceeded our predefined goals for feasibility, acceptability and utility.

#### Feasibility: capacity to engage members of the public

At each “PPI Hawker” more than 20 members of the public were engaged, and within each session most individuals (85%) responded to each of the questions posed (these are listed in Appendices 1 and 2). The “PPI Hawker” concept was well received by the public; 96 members of the public were approached, whilst 72 took part in one of the three sessions. In other words, 75% of those approached at their tables agreed to engage in a conversation with us.

#### Acceptability: the public’s perspective of the “PPI Hawker”

We involved members of all three ethnic groups in Singapore and spoke to adults who appeared to be from a wide age range, confirming accessibility of the “PPI Hawker”.

From the public’s feedback the opportunity to take part in the “PPI Hawker” conversations was appreciated, supporting “PPI Hawker”’s acceptability. Most members of the public we spoke to were “*happy to give [us their] opinion*” and to informally share with us their thoughts on the “PPI Hawker”. Participants enjoyed *“feeling helpful*” contributing to something that would benefit their community. They felt that by meeting in the familiar space of the hawker centres, researchers lost “*their authoritarian attitude*”. Moreover, they recognised that “*researchers don’t know [our needs and wants], we have to tell them*”. The format of the “PPI Hawker” as an involvement method for Singaporeans was widely supported; participants valued its concise and one-off nature, because of the pressures on their time and no wish to talk about research on a regular basis. One member of the public finished their conversation with us by thanking us for meeting the public: “*You are doing a very good thing for people [coming to ask for people’s perspectives at the hawker centre]. I have to appreciate*”.

#### Utility: population-based study’s researchers’ feedback

Results of the “PPI Hawker” were presented to the study’s Steering Group, composed of the 6 Study Principal Investigators, the Operations Manager, the Recruitment Manager and occasionally other researchers from the team. The Steering Group’s response to the feedback from the public about their population-based study was encouraging, and they expressed the need to continue with the “PPI Hawkers” to also understand the views of populations from different neighbourhoods. The researcher involved as a facilitator commented on how “*The public’s opinion are very diverse, and I learnt that if you spend the time to listen to public, there are many things that you can learn. People are generally willing to help, and their feedback will only make our research stronger!*” With subsequent “PPI Hawkers” her appreciation of their value was strengthened by observing the hidden knowledge within different communities. She commented: “*An eye opener, certainly I could see the totally different perspective from those of lower socio-economic classes about research health screening*”.

The discussion of the feedback from participants prompted refinements to the “PPI Hawker” including more consistency in the terminology used. For example, while some facilitators had used the word “confidentiality” others had used “anonymity” when discussing data sharing issues. This inconsistency made the PPI feedback less useful as it was not always possible to know which term the pubic were responding to. As a result, we agreed facilitator’s should only use the terminology on the cue cards and would always explain any technical terms before engaging in discussion.

Examples of how PPI impacted on the design of the population-based study can be found in the Table 2 below. Researchers considered the feedback provided by “PPI Hawker” participants for all questions and intend to incorporate some aspects for each of the issues discussed. Some of those have already been operationalised, as discussed in Table 2.

**Table 2:** Impact of the “PPI Hawkers” on the population-based study design

After the “PPI Hawker” Pilot, members of the Steering Group were receptive to the public’s suggestions to allay concerns about data security by sharing more information on how participant data is stored and by requesting additional consent for data sharing with commercial collaborators (Question 1), to consider how the research data could be incorporated into the individual’s health records (Question 2) and to regularly update participants on the study’s progress (Question 3).	
After the two subsequent “PPI Hawkers” the researchers on the population-based study introduced a number of changes to the research process in response to the public’s comments and support. Researchers began establishing new recruitment strategies, by formalising agreements with various employers (Question 1) and setting up a community Advisory Group to provide regular and more detailed advice on matters arising as the project progresses. (Question 3). Further changes included amendments to the informed consent form:	
i) An improved explanation of the term ‘incidental findings’, using simpler terms and detailing the pros and cons of being informed of these abnormal results.	
ii) Giving study participants the option to decide whether they would like to have their incidental findings communicated to them or not (Question 4).	
iii) Including a question relating to data sharing. It was decided to offer research participants at initial recruitment the opportunity to choose who in the future their data could be shared, thus negating the need to recontact them (Question 2). The four options to select from are (i) Universities/ academic institutions, (ii) Health institutions, (iii) Government institutions and/or (iv) Commercial bodies.	

## Discussion

### Summary

In three “PPI Hawkers” we were able to engage 72 members of the public in discussions about issues the researchers of a population-based study wanted the public’s perspective. This novel approach of involving lay people on research-related matters achieved an engagement rate of 75%. Participants came from the three main ethnic groups in Singapore and appeared to be broad in age. Since the hawker centres are spaces visited by almost all Singaporeans, this PPI method facilitates engagement with a broad sector of society in different neighbourhoods. Using short one-off conversations in their mother tongue in a familiar environment the majority of participants were willing to discuss all of the questions posed by the researchers. Researchers recognised the utility of the “PPI Hawker”, reflecting on people’s willingness to consider research issues, engaging in informative conversations and ask relevant questions. During these conversations, facilitators observed the traditional barriers between experts and non-experts being broken down, leading to mutual respect and sharing of ideas.

#### PPI facilitator’s experience

Facilitators observed how the informal setting allowed members of the public to talk openly and freely share personal experiences. This relaxed environment allowed us to approach audiences unfamiliar with research, where they felt able to contribute and also to ask questions about other topics beyond those suggested by the researchers. Facilitators were encouraged by their experiences of the “PPI Hawkers”, and highlighted the feasibility and accessibility of this method of involvement in the Singaporean context.

The reflexive notes of the “PPI Hawker” team were universally encouraging, sometimes describing characteristics that exceeded expectations:

*“I was surprised by how most people were receptive and keen to discuss the project with us. Singaporeans are often zealous in the protection of their privacy and are reluctant to share their opinions. However, those we interacted with were willing to talk at length about the issues, curious about the research, and seemed to enjoy the experience”* (“PPI Hawker” co-ordinator).

One facilitators recorded how the public’s initial hesitancy to be involved was quickly transformed into engagement:

*“People were apprehensive when we first approached them (like we were going to sell something/ask them for something/like we were disturbing quiet, family time) but once it was clear we just wanted their OPINIONS, the conversation flowed quite effortlessly. I was surprised to see them have so much to say, so much to contribute to each of the questions asked. The public are not as oblivious or ill-informed as some may think, they may not be researchers but everything that was said made perfect sense and was of great value. We could not have predicted those responses or gotten a more honest feel for their perception of research in general or the barriers and facilitators to research if we did not sit down and talk to them in person, in a natural setting as we did today.” (Public facilitator, reflecting on sessions #2 and #3)*

### Advantages of the “PPI Hawker”

The “PPI Hawkers” have the potential to be used more widely for PPI in Singapore, and potentially in other Asian contexts. This approach to PPI has identified two major attributes:

Firstly, the “PPI Hawker” reduces communication barriers, making it easier for researchers to interact with the public and vice versa. The process of the public inviting researchers to join them at their own tables appeared to enable the public more control over the encounter, and guide the amount and direction of the discussion. These conversations become opportunities to share knowledge and work together to improve the research design. We observed within the “PPI Hawker” dialogue, reciprocity, openness and respect, enabling thoughtful communication between the facilitators and the public.

Secondly the “PPI Hawker” reduces the barriers between experts and the public, flattening or reversing the pyramid of hierarchy [51]. Conventionally lay people are reluctant to challenge perceived expertise [52, 53], but the round table discussions appeared to generate mutual trust, and a willingness for the non-expert to comment, critique and challenge the research. Such exchange is often difficult to achieve in very hierarchical societies, like Singapore [52, 54] but the “PPI Hawker” appears to achieve this by the development of a collective consciousness.

### Effective and efficient use of resources

PPI is often characterised as a time and energy intensive activity, and it can be this that deters researchers’ enthusiasm and engagement for PPI [6]. The “PPI Hawker” addresses some of these concerns as it does not require the administrative burden or the expenses of sending out invitations, hiring a venue or transport for participants. However, it does require time for the initial planning (a one-off activity), and then the conduct of each “PPI Hawker” and the collating of ideas. In our experience the time spent planning preparing and training for the project-specific PPI was 10 person hours in total. Each “PPI Hawker” required 2 h of facilitators time (including a 30-min briefing in advance). The final collation of comments and summarising them for the research team required a further 8 h of the coordinator’s time and 1 h for each of the other facilitators’ time.

### Limitations and strengths of the “PPI Hawker”

A challenge for PPI globally is the need to hear a wide diversity of perspectives. Increasingly more groups have been empowered and are being heard, but some groups still remain at the margins. Reluctance to participate in PPI [6] is known to be more challenging to those with poor research knowledge, lower literacy and limited oral skills, characteristics often seen within those sub-populations who have the greatest health needs. We found the presence of the public facilitators was particularly helpful with developing trust, for example with the Indian population an introduction in Tamil defused feelings of being caught in an unfamiliar situation to inclusion in a conversation in which they were central.

“PPI Hawkers” offer a helpful solution to this challenge, but they are not without their limitations. For example, it is recognised that long-term involvement throughout the different stages of a research project increases the impact of PPI [7] but the “PPI Hawker” is a one-off encounter. Not collecting personal and demographic data from participants makes it difficult to characterise them exactly. For example, we wanted an ethnic mix and used appearance to identify the ethnic group of participants. Using appearance may not always be adequate, for example if one wishes to target people of a specific age, sexuality, marital status or income group. Lastly, the “PPI Hawker” may not be appropriate for all types of research, including studies about sensitive topics or complex studies where substantial background and explanation are needed before the public are able to comment.

Finally, alongside positive aspects of the “PPI Hawker”, some negative views were also noted. Around a quarter of members of the public approached wanted to be “left alone”, others wished to discuss topics not related to the research study (for example, digressing to complain about the government). However, only one individual responded with some aggression, sharing their dogmatic beliefs about medicine. None of our offers of a drink were accepted, and occasionally a participant expressed mild offense when offered a free drink, they considered it as too small a token of appreciation, because it was something that they could easily afford for themselves.

We have demonstrated the feasibility of the “PPI Hawkers”, a novel approach suitable in a non-western culture, complementing widely used PPI Cafes and User Groups. Although our experience is currently confined to Singapore we anticipate that this model of involvement would be applicable to other Asian countries where the traditional concept of public food markets are also commonplace.

As a potential refinement of the “PPI Hawkers” may be the use of some advanced publicity of the event, rather than descending unannounced on diners. This may have reduced the suspicion some people expressed and confirm our legitimacy. We are also keen to explore in the future whether the “PPI Hawker” could be an avenue for dissemination of research findings, an under-developed aspect of PPI [55]. Research findings continue to be shared conventionally, disseminated in academic environments (for example, conference presentations, journal papers and academic books) and far less through channels easily accessible for the public (for example, public exhibitions, podcasts and blogs). “PPI Hawkers” may be an effective way to increase the public’s awareness of the findings of health-related research that could impact on their lives. The feasibility of this needs further exploration and piloting.

## Conclusion

This study has demonstrated how the “PPI Hawker” aligns with the ethos of public involvement in research. It has the potential to be the major model for PPI initiatives in Singapore, facilitating knowledge exchange in community spaces accessible to a wider number of people. Further feasibility studies are needed to confirm the “PPI Hawkers” utility when used to discuss studies in different topic areas and with different methods, but preliminary findings are encouraging.

## Data Availability

The datasets generated during and/or analysed during the current study are available from the corresponding author on reasonable request.

## Appendix 1

### Questions for the public in the “PPI Hawker” pilot

1. If you were a participant in the study…
  - What would be needed for you to be reassured that your personal data is being protected?
  - What types of information are acceptable to collect from people participating?
  - Would it be acceptable for anonymized data to be shared with:
    - Other health care professionals in Singapore?
    - Industries in Singapore?
    - Overseas organisations?
2. Increasingly health records are being digitalized: in theory personal health records and the research study could be linked, and data shared…
  - What do you think the benefits could be of such data linkage?
  - What concerns might you have of researchers from this study accessing (*taking data*) your electronic health data or contributing (*providing data*) to your electronic health data?
3. If in the future this study asked its participants to return for further data collection…
  - What concerns might people have about continuing involvement?
  - How could these concerns be reduced?

## Appendix 2

### Questions for the public in “PPI Hawker” sessions 2 and 3

1. If you were to be recruited for this study...
  - What would be attractive in this study?
  - How would you like to be recruited?
  - What concerns would you have about it?
  - How could these concerns be addressed?
2. Research records are anonymised, so the researchers don’t know participants’ names and personal information. The information researchers work with is called anonymised research data.
  - How would you feel if your anonymised research data was shared with:
  - Other healthcare professionals in Singapore?
  - Other researchers in Singapore?
  - Public institutions in Singapore?
  - Commercial partners in Singapore?
    - Then ask the same for overseas.
3. The study has a commitment with the communities it serves and would like to engage its participants further, even after their visit. Previous participants are part of the study “family”, and we want them to feel included. By building long-term partnerships with them they can advise on how to develop the study and share their expectations.
  - Would you like to be engaged with the study after your visit?
  - If so, how often? For how long? With whom?
4. 10 years after the study has finished, researchers find a gene in a research participant which might indicate increased risk of cancer later on in life (this is known as an incidental finding). However, treatment for this cancer still does not exist.
  - Do you think the study should track back the participant and inform them of this finding?
  - Would you think differently if there was some form of treatment available?
  - Do you have any concern/ anxiety knowing that this could happen?
  - What sort of incidental finding do you think should be reported back to participants?

## Abbreviations

PPI: Patient and public involvement
PERC: Patient Experience Research Centre
